# Development of aortic valve stenosis in myeloperoxidase antineutrophil cytoplasmic antibody-associated vasculitis with renal involvement

**DOI:** 10.1371/journal.pone.0245869

**Published:** 2021-01-22

**Authors:** Midori Hasegawa, Jin Iwasaki, Satoshi Sugiyama, Takuma Ishihara, Yoshihiro Yamamoto, Hiroaki Asada, Shigehisa Koide, Hiroki Hayashi, Kazuo Takahashi, Daijo Inaguma, Yukio Yuzawa, Naotake Tsuboi

**Affiliations:** 1 Department of Nephrology, Fujita Health University School of Medicine, Toyoake, Aichi, Japan; 2 Tokoname City Hospital, Tokoname, Aichi, Japan; 3 Kanayama Clinic, Nagoya, Aichi, Japan; 4 Gifu University Hospital Innovative and Clinical Research Promotion Center, Gifu City, Gifu, Japan; 5 Toyota Memorial Hospital, Toyota, Aichi, Japan; 6 Okazaki City Hospital, Okazaki, Aichi, Japan; 7 Department of Biomedical Molecular Sciences, Fujita Health University School of Medicine, Toyoake, Aichi, Japan; 8 Department of Internal Medicine, Fujita Health University Bantane Hospital, Nagoya, Japan; University of Mississippi Medical Center, UNITED STATES

## Abstract

**Introduction:**

Degenerative aortic valve stenosis (AS) is a chronic progressive disease that resembles atherosclerosis development. Antineutrophil cytoplasmic antibody-associated vasculitis (AAV) is reportedly associated with accelerated atherosclerosis. This study aimed to examine the development of AS in patients with myeloperoxidase-AAV (MPO-AAV) with renal involvement at more than 1 year after the onset of vasculitis.

**Methods:**

We performed a retrospective review of clinical records of MPO-AAV patients with renal involvement without AS at the onset of vasculitis who were treated in three hospitals and three dialysis clinics.

**Results:**

The study included 97 MPO-AAV patients with renal involvement and 230 control patients with chronic kidney disease (CKD). Among them, 64 patients had AS. The prevalence rates of AS were 28.9% and 15.7% in MPO-AAV and control patients, respectively (p = 0.006). The multivariable logistic regression analysis showed that MPO-AAV, dialysis dependence, and hypertension were independently associated factors for AS. In MPO-AAV patients, systolic blood pressure was positively significantly associated with AS, whereas glucocorticoid dose of induction therapy was negatively significantly associated. The use of cyclophosphamide tended to be negatively associated with AS. The survival rate was significantly lower for patients with AS than for those without AS.

**Conclusions:**

The AS prevalence rate was significantly higher in MPO-AAV patients at more than 1 year after the onset of vasculitis than in control CKD patients. Therefore, regular monitoring of echocardiography during MPO-AAV treatment is suggested.

## Introduction

Antineutrophil cytoplasmic antibody (ANCA)-associated vasculitis (AAV) is a form of necrotising vasculitis with few immune deposits, predominantly affecting small vessels, and it is associated with ANCA specific for myeloperoxidase (MPO) or proteinase 3 [1]. The kidney is the most affected organ, followed by the lung, ear, nose, throat, nervous system, and cutaneous tissues [2]. A meta-analysis indicated that AAV presents a relative risk of 1.65 (95% confidence interval [CI]: 1.23–2.22) in all cardiovascular events [3], and the risk substantially stems from an increase in the occurrence of ischaemic heart disease [4]. Atherosclerosis is one of the main pathophysiological mechanisms of ischaemic heart disease [5], and degenerative aortic valve stenosis (AS) is a chronic progressive disease that resembles atherosclerosis development [6]. AAV has been reported to be associated with accelerated atherosclerosis [7, 8]. Epidemiological studies have demonstrated that hypertension, diabetes, dyslipidaemia, chronic kidney disease (CKD), and ageing are associated with degenerative AS [9, 10]. MPO-ANCA-associated vasculitis (MPO-AAV) patients often have some of these AS-associated factors, including CKD [2]. CKD with AS was associated with higher cardiac and all-cause mortality rate than CKD without AS [11]. This study aimed to examine the development of AS (atherosclerotic lesion) in MPO-AAV patients with renal involvement at more than 1 year after the onset of vasculitis and assess the effect of AS on long-term prognosis.

## Materials and methods

### Study population

**Fig 1:**
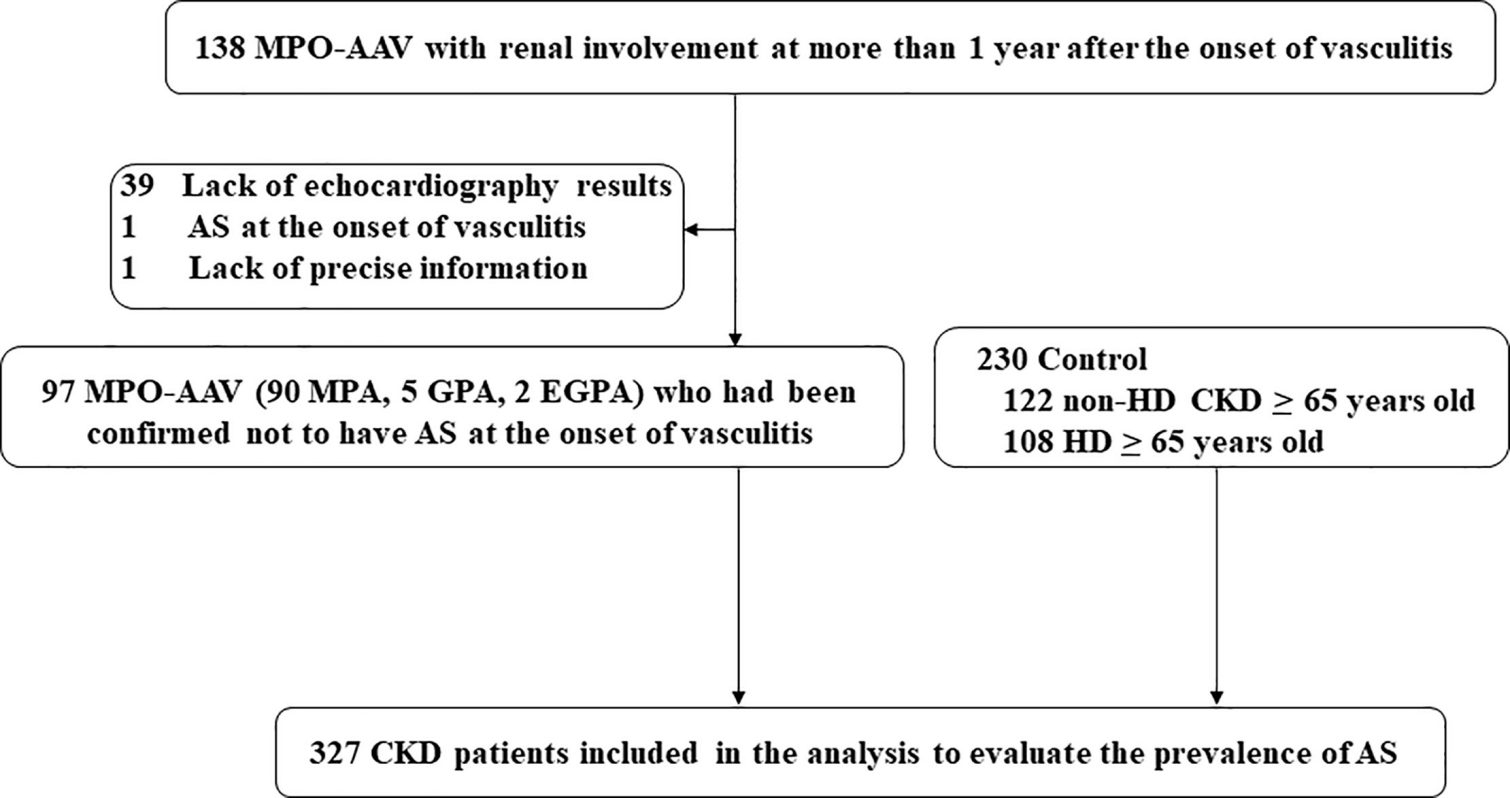
Patient flow. MPO-ANCA, myeloperoxidase antineutrophil cytoplasmic antibody; MPO, microscopic polyangiitis; GPA, granulomatosis with polyangiitis; EGPA, eosinophilic granulomatosis with polyangiitis; AS, aortic valve stenosis; CKD, chronic kidney disease; HD, haemodialysis.

The eligible patients fulfilled the following criteria: 1) were MPO-ANCA-positive at diagnosis; 2) had surrogate markers for renal vasculitis, i.e. haematuria associated with red cell casts, dysmorphic erythrocytes, or haematuria (2+) and proteinuria (2+) on urinalysis; 3) had undergone transthoracic echocardiography at 1 year or more after the onset of vasculitis; and 4) had been treated at the Fujita Health University Hospital, Department of Nephrology from January 2005 to December 2017 or could have been examined in December 2016 at the following institutions: Okazaki City Hospital, Kanayama Clinic, Tokai Clinic, Toyota Memorial Hospital, and Nishio Clinic. Patients not requiring renal replacement therapy and those who underwent this therapy at the last visit were regarded as dialysis-independent and dialysis-dependent patients, respectively. The patients 1) who had undergone aortic valve replacement surgery or experienced AS at the onset of MPO-AAV; 2) who could not be followed up within 1 year after the onset of vasculitis; or 3) whose medical records could not be confirmed at the time of echocardiography (Fig 1) were excluded. As MPO-AAV occurs in elderly individuals on an average [2], aged patients (≥ 65 years) were chosen as controls. Aged patients who had undergone preoperative echocardiography within 3 months before surgery at Fujita Health University Hospital between July 2014 and December 2016 and had a history of consulting a nephrologist due to impaired renal function served as controls for dialysis-independent patients. Those who were scheduled for surgery for valvular heart disease were excluded. In contrast, aged patients who had undergone maintained haemodialysis for over 4 months at Fujita Health University Hospital or Kanayama Clinic served as controls for dialysis-dependent patients.

### Ethics

All procedures performed in studies involving human participants were in accordance with the ethical standards of the institutional and/or national research committee and with the 1964 Helsinki declaration and its later amendments or comparable ethical standards. This study was approved by the Ethics Committee of Fujita Health University School of Medicine (authorised number: MH17-020). Informed consent was waived due to the retrospective nature of the study.

### Data collection

Data were analysed retrospectively based on medical records. Obtained patient data included age, sex, blood pressure, Birmingham Vasculitis Activity Score (BVAS) [12], Japanese clinical grade of rapidly progressive glomerulonephritis [13], serum phosphorus, corrected Ca, C-reactive protein (CRP), lipid, MPO-ANCA titre (measured using enzyme-linked immunosorbent assay until October 2012 and chemiluminescence enzyme immunoassay after November 2012), serum creatinine, dialysis history, glucocorticoid dose (conversion to prednisolone), cyclophosphamide usage, presence of diabetes mellitus, and survival outcomes. Microscopic polyangiitis (MPA), granulomatosis with polyangiitis (GPA), and eosinophilic granulomatosis with polyangiitis (EGPA) were defined according to the European Medicines Evaluation Agency algorithm [14]. Patients were classified as having diabetes when their medical records contained documentation of a history of diabetes, diagnosis of diabetes on admission, or use of an oral antihyperglycemic agent or insulin. Hypertension was defined as having systolic blood pressure ≥140 mmHg, diastolic blood pressure ≥90 mmHg, or antihypertensive drug administration.

### Definition of AS

With reference to the American College of Cardiology and American Heart Association Valvular Heart Disease Guidelines in 2014 [15], a case of leaflet calcification of a valve with some reduction in systolic motion and aortic maximum velocity (Vmax) >2.0 m/s, mean delta pressure gradient >20 mmHg, or aortic valve area (AVA) <1.0 cm^2^ was defined as AS. According to the same guideline, Vmax values of 2.0–2.9, 3.0–3.9, 4.0–4.9, and >5.0 m/s were classified as mild, moderate, severe, and very severe cases of AS, respectively.

### Statistical analysis

For analyses of clinical characteristics of MPO-AAV and control patients, continuous variables are presented as median and interquartile range (IQR), whereas categorical variables are presented as frequencies. Continuous and categorical variables were compared using the Mann–Whitney U and using contingency tables and the chi-square test, respectively. In the primary analysis, multivariable logistic regression analysis was performed to assess the effect of MPO-AAV on the prevalence of AS with adjustment for age, sex, dialysis dependence, hypertension, and low-density lipoprotein (LDL). Given the number of AS (64 events) cases, we limited the logistic regression model to 5 covariates to avoid overfitting [16, 17]. Missing covariates included in the regression model were imputed using multiple imputation methods. For secondary analysis, to assess the associations between AS and clinical factors in MPO-AAV, a subgroup analysis was conducted using a logistic regression model with adjustment for dialysis dependence that included only MPO-AAV patients. Because the number of AS cases was 28 in the MPO-AAV patients, the covariate was limited to dialysis dependence exclusively. Laboratory data at the onset of MPO-AAV were used for this logistic regression analysis. Additionally, to evaluate the effect of AS on mortality in MPO-AAV patients, Simon and Makuch’s modified Kaplan–Meier estimation and time-varying Cox proportional hazard analysis adjusted for covariates (age and diabetes) were performed. The occurrence of AS was treated as a time-dependent variable. All statistical inferences were performed using a two-sided significance level of 5%, and data management and analyses were performed using R statistical software (version 4.0.2; R Foundation for Statistical Computing, Vienna, Austria).

## Results

### Patients’ characteristics

Of 138 MPO-AAV patients, 39 did not have echocardiography results at 1 year after or at the onset of vasculitis; one patient experienced AS at the onset of vasculitis, and another patient did not have precise information. The remaining 97 AAV (92.8% MPA, 5.1% GPA, and 2.1% EGPA) and 230 CKD patients as controls were registered in this study (Fig 1). Table 1 shows the patient characteristics and laboratory data at the time of echocardiography. The median age was 74 years (IQR: 67–79 years) in MPO-AAV patients and 74 years (IQR: 69–79 years) in control patients. Moreover, 50.5% and 68.7% of MPO-AAV and control patients were men, respectively. The proportion of patients who were smokers, had diabetes, and hypertension, and were dialysis-dependent was 38.1%, 28.9%, 82.1%, and 40.2% in the MPO-AAV group, and 54.0%, 36.5%, 78.7%, and 47.0% in the control group, respectively. Regarding the laboratory data, no significant differences were observed between MPO-AAV and control patients with respect to hemoglobin, albumin, creatinine, and CRP levels. The LDL, and HDL-cholesterol, and calcium levels were significantly higher in MPO-AAV patients than in control patients. In contrast, phosphate levels was significantly higher in control patients than in MPO-AAV patients.

**Table 1:** Clinical characteristics of MPO-AAV and control patients.

	Control (n = 230)	MPO-AAV (n = 97)	All (n = 327)	p-value
Age (years)	74 (69, 79)	74 (67, 79)	74 (68, 79)	0.34
Male (n, %)	158 (68.7%)	49 (50.5%)	207 (63.3%)	0.002
BMI	21.0 (18.4, 23.3)	20.1(18.0, 22.4)	20.6 (18.3, 23.0)	0.11
Systolic BP (mmHg)	133 (119, 149)	133 (124, 145)	133 (121, 147)	0.53
Diastolic BP (mmHg)	72 (63, 81)	73 (66, 80)	72 (64, 80)	0.63
Smoking (n, %)	101 (54.0%)	32 (38.1%)	133 (49.1%)	0.018
Comorbidities				
Diabetes (n, %)	84 (36.5%)	28 (28.9%)	112 (34.3%)	0.20
Hypertension (n, %)	181 (78.7%)	78 (82.1%)	259 (79.7%)	0.55
Dialysis dependence (n, %)	108 (47.0%)	39 (40.2%)	147 (45.0%)	0.28
Dialysis vintage (month)	88 (40, 184)	46 (16, 82)	71 (37, 163)	0.002
Laboratory data				
Hemoglobin (g/dL)	11.1 (10.2, 11.8)	11.3 (10.2, 12.9)	11.1 (10.2, 12.0)	0.23
Albumin (g/dL)	3.6 (3.2, 3.9)	3.6 (3.3, 4.0)	3.6 (3.2, 3.9)	0.39
LDL-C (mg/dL)	93 (72, 111)	102 (82, 126)	96 (74, 115)	0.003
HDL-C(mg/dL)	45 (35, 56)	70 (55, 82)	50 (39, 65)	<0.001
Creatinine (mg/dL) *	1.38 (1.01, 2.19)	1.40 (1.09, 2.07)	1.39 (1.03, 2.17)	0.69
CRP (mg/dL)	0.17 (0.15, 0.56)	0.15 (0.15, 0.50)	0.15 (0.15, 0.50)	0.096
P (mg/dL)	4.3 (3.4, 5.5)	3.8 (3.2, 4.6)	4.1 (3.4, 5.2)	0.001
Ca (mg/dL)	8.9 (8.4, 9.4)	9.1 (8.6, 9.5)	9.0 (8.5, 9.4)	0.018

Continuous variables are presented as median and interquartile range, and categorical variables are presented as numbers and frequencies.

*Dialysis-dependent cases were excluded.

Abbreviations: MPO-AAV, myeloperoxidase antineutrophil cytoplasmic antibody-associated vasculitis; BMI, body mass index; BP, blood pressure; LDL-C, low-density lipoprotein cholesterol; HDL-C, high-density lipoprotein cholesterol; CRP, C-reactive protein.

### The prevalence of AS

We summarised the prevalence of AS by severity. The proportion was significantly higher in MPO-AAV patients (28.9%) than in control patients (15.7%) (p = 0.006) (Table 2); 60.7%, 14.3%, and 25.0% of AS patients with MPO-AAV and 75.0%, 16.7%, and 8.3% of control patients were classified into the mild, moderate, and severe categories, respectively.

**Table 2:** Prevalence of aortic valve stenosis.

AS severity	Control (n = 230)	MPO-AAV (n = 97)	All (n = 327)	p-value
The number of AS (%)	36 (15.7%)	28 (28.9%)	64 (19.6%)	0.006
AS severity				0.19
Mild	27 (75.0%)	17 (60.7%)	44 (68.8%)	
Moderate	6 (16.7%)	4 (14.3%)	10 (15.6%)	
Severe	3 (8.3%)	7 (25.0%)	10 (15.6%)	

Abbreviations: AS, aortic valve stenosis; MPO-AAV, myeloperoxidase antineutrophil cytoplasmic antibody-associated vasculitis.

### Multivariable logistic regression analysis for AS

The results of multivariable logistic regression analysis for AS are shown in Table 3. MPO-AAV, dialysis dependence, and hypertension were classified as independent associated factors for AS. In the other models wherein diabetes, HDL-C, Ca, or P was a variable instead of LDL-C, MPO-AAV, dialysis dependence, and hypertension were similarly significant factors (S1–S4 Tables).

**Table 3:** Multivariable logistic regression analysis for aortic valve stenosis in 327 CKD patients.

	OR	95% LCI	95% UCI	p-value
MPO-AAV (yes = 1)	2.89	1.51	5.54	0.001
Dialysis dependence (yes = 1)	7.35	3.61	14.99	<0.001
Age at echocardiography (per 1-year increase)	1.01	0.97	1.05	0.80
Sex (Male)	1.83	0.94	3.55	0.075
Hypertension (yes = 1)	3.48	1.28	9.48	0.015
LDL-C (per 1 mg/dL increase)	1.00	0.99	1.02	0.37

MPO-AAV, myeloperoxidase antineutrophil cytoplasmic antibody-associated vasculitis; CKD, chronic kidney disease; LDL-C, low-density lipoprotein cholesterol; OR, odds ratio; LCI, lower confidence interval; UCI, upper confidence interval.

### Associated factors for AS in MPO-AAV patients

Vasculitis classification, severity, and treatment in MPO-AAV patients are listed in Table 4. At the last echocardiography, 94.8% were in remission, as indicated by the zero BVAS. To evaluate the associated factors for AS in MPO-AAV patients, logistic regression analysis for AS with adjustment for dialysis dependence was performed (Table 5). Systolic blood pressure was positively significantly associated with AS, whereas glucocorticoid dose of induction therapy was negatively significantly associated. The use of cyclophosphamide tended to be negatively associated with AS.

To confirm the relationship between the observational period from MPO-AAV onset to echocardiography and AS development, we compared the duration of observation between patients with and without AS. For patients with AS, we defined the first confirmation of AS after the onset of MPO-AAV as the end of observation. In contrast, for patients without AS, we defined the date of the most recently performed echocardiography as the end of observation. No significant difference in the duration of observation was observed between patients with and without AS (Table 6).

**Table 4:** Vasculitis classification, severity, and treatment in MPO-AAV patients.

MPA/GPA/EGPA (n, %)	90 (92.8%)/5 (5.1%)/2 (2.1%)
Age at onset of vasculitis	68 (61–75)
BVAS at onset	17 (13, 20)
RPGN clinical grade at onset (n, %)	Ⅰ 23 (25.6%), Ⅱ 43 (47.8)
	Ⅲ 19 (21.1%), Ⅳ 5 (5.5%)
MPO-ANCA at onset of vasculitis	
ELISA (EU) (n = 69, Until October 2012)	238.0 (95.5, 448.5)
CLEIA (U/mL) (n = 24, After November 2012)	134.0 (67.5, 134.0)
MPO-ANCA at echocardiography	
ELISA(EU) (n = 24, Until October 2012)	11.5 (5.0, 63.0)
CLEIA (U/mL) (n = 65, After November 2012)	4.3 (0.8, 12.0)
Glucocorticoid dose of induction therapy (mg/day)	40 (30, 40)
Glucocorticoid dose at echocardiography (mg/day)	8 (5, 11)
Use of cyclophosphamide (n, %)	34 (35.1%)
Follow up duration from vasculitis onset	
to the last echocardiography (month)	57 (27, 105)
to the last visit (month)	80 (43, 124)

Abbreviations: MPO-AAV, myeloperoxidase antineutrophil cytoplasmic antibody-associated vasculitis, MPA, microscopic polyangiitis; GPA, granulomatosis with polyangiitis; EGPA, eosinophilic granulomatosis with polyangiitis; BVAS, Birmingham Vasculitis Activity Score; RPGN, rapidly progressive glomerulonephritis; MPO-ANCA, myeloperoxidase antineutrophil cytoplasmic antibody; ELISA, enzyme-linked immunosorbent assay; CLEIA, chemiluminescent enzyme immunoassay.

Glucocorticoid dose is expressed as conversion to prednisolone.

Continuous variables are presented as median and interquartile range, and categorical variables are presented as numbers and frequencies.

**Table 5:** Logistic regression analysis for aortic valve stenosis in MPO-AAV patients.

	OR	95% LCI	95% UCI	p-value
Age at onset of vasculitis (per 1-year increase)	1.02	0.98	1.08	0.32
Sex (Male)	1.40	0.51	3.88	0.52
BVAS at onset	0.92	0.82	1.04	0.172
RPGN clinical grade at onset	1.25	0.65	2.41	0.51
Glucocorticoid dose of induction therapy per 1mg/day increase	0.94	0.90	0.99	0.016
Glucocorticoid dose at echocardiography per 1mg /day increase	1.06	0.97	1.16	0.22
Use of cyclophosphamide (yes = 1)	0.30	0.09	1.01	0.052
Smoking (yes = 1)	1.19	0.36	3.92	0.77
Diabetes (yes = 1)	1.18	0.38	3.65	0.77
Systolic BP per 10 mmHg increase	1.04	1.01	1.08	0.02
Diastolic BP per 10 mmHg increase	1.04	0.99	1.09	0.11
Hb per 1 g/dL increase	0.85	0.63	1.14	0.28
Alb per 1 g/dL increase	0.85	0.35	2.10	0.73
P per 1 mg/dL increase	1.13	0.74	1.73	0.58
Ca per 1 mg/dL increase	0.51	0.20	1.31	0.16
LDL-C per 1 mg/dL increase	1.00	0.97	1.02	0.82
HDL-C per 1 mg/dL increase	0.97	0.91	1.03	0.33

*All logistic regression analyses were performed adjusting for dialysis dependence.

Glucocorticoid dose is expressed as conversion to prednisolone.

Abbreviations: MPO-AAV, myeloperoxidase antineutrophil cytoplasmic antibody-associated vasculitis; BP, blood pressure; Hb, hemoglobin; Alb, albumin; HDL-C, high-density lipoprotein cholesterol; LDL-C, low-density lipoprotein cholesterol; OR, odds ratio; LCI, lower confidence interval; UCI, upper confidence interval; BVAS, Birmingham Vasculitis Activity Score; RPGN, rapidly progressive glomerulonephritis.

**Table 6:** Mann Whitney U test for the period from MPO-AAV onset to echocardiography.

	Non-AS, N = 263	AS, N = 64	p-value
The period (month)	52.0 (26.0, 94.0)	63.4 (43.0, 116.5)	0.35

The values are presented as median and interquartile range.

Abbreviations: AS, aortic valve stenosis; MPO-AAV, myeloperoxidase antineutrophil cytoplasmic antibody-associated vasculitis.

### Overall survival in MPO-AAV patients

**Fig 2:**
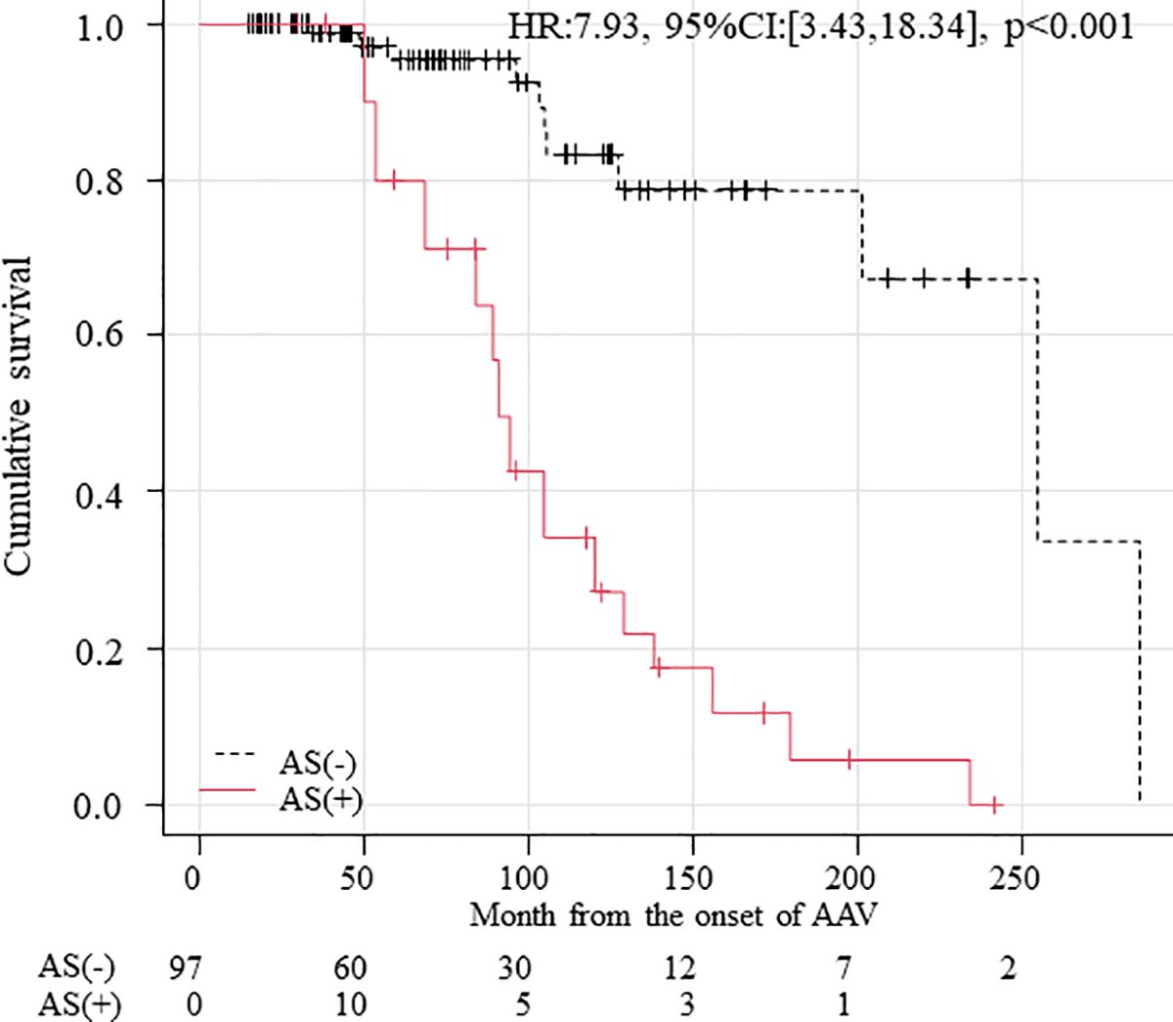
Simon and Makuch’s modified Kaplan–Meier curve of survival in MPO-ANCA-associated vasculitis patients. The survival rate was significantly lower for patients with aortic valve stenosis than for those without aortic valve stenosis (p < 0.001). Time-varying Cox proportional hazard analysis was adjusted for age and diabetes. AS, aortic valve stenosis.

Twenty-five patients, including 14 AS patients in the MPO-AAV group, died during an 80 (IQR: 43–124)-month observation period. The causes of death in 14 AS patients included infection (eight patients), heart failure (three patients), gangrene of a leg (two patients), and haemorrhagic shock (one patient). Simon and Makuch’s survival curve was shown in Fig 2. The survival rate of patients with AS was significantly lower than that of patients without AS (hazard ratio [HR]: 7.93; 95% CI: 3.43–18.34; p <0.001).

## Discussion

In our cohort, 64 patients (19.6%) had AS, comprising MPO-AAV patients who were followed up for more than 1 year and patients aged ≥65 years with CKD. As we aimed to examine the development of AS during MPO-AAV treatment, we excluded the patients who had AS at the onset of vasculitis. We selected 6 categorical variables of age, sex, hypertension, LDL-cholesterol, dialysis and MPO-AAV for multivariable logistic analysis. Age and sex are basic information, whereas hypertension and dyslipidaemia are the factors associated with AS [9]. The other factors associated with AS, including HDL-C, diabetes [9], phosphorus [18], and calcium [19], were separately included in the multivariable logistic analysis. The presence of MPO-AAV, dialysis dependence, and hypertension were independently associated factors for AS among the 6 categories. LDL-cholesterol levels were significantly higher in MPO-AAV than in control patients. Deterioration of lipid control might accelerate the development of atherosclerosis in MPO-AAV patients. Blood pressure was associated with AS in our cohort, as in the previous reports, in the general population [20].

Glucocorticoids have been demonstrated to be associated with aortic valve calcification [21]. In rheumatoid arthritis, cumulative glucocorticoid exposure was associated with increased cardiovascular mortality [22]. In this cohort, the median dose of glucocorticoid was 8 mg/day at median 57 months after the onset of MPO-AAV when echocardiography was performed. The metabolic effects of glucocorticoids [23] might be related to AS development. In the Japanese clinical practice guidelines for rapidly progressive glomerulonephritis, treatment algorithm was shown according to the clinical grade [13]. Cyclophosphamide was mandatory only for patients with clinical grades III or IV, <70 years of age, and not dialysis-dependent. The rate of cyclophosphamide use was 35.1% in this cohort. Odds ratio of the use of cyclophosphamide for AS was 0.30 [0.09–1.01, p = 0.052]. The possibility of increased cumulative dose of glucocorticoids by glucocorticoid monotherapy compared with the combined use of immunosuppressants or biological agents are important issues. The initial glucocorticoid dose was also associated with AS. The attending physicians might refrain from strong immunosuppression due to the patients’ comorbidities. This might be associated with the risk of developing AS, and the inadequate initial treatment might then lead to the increased cumulative glucocorticoid dose.

The EULAR/ERA-EDTA guidelines recommended that remission maintenance therapy for AAV should be continued for at least 24 months following the induction of sustained remission [24]. Regarding relapse, a meta-analysis showed that the proportion of patients with relapse was 14% (95% CI: 10–19%) and 43% (95% CI: 33–52%) in non-zero and zero glucocorticoid target dose studies, respectively [25]; thus, glucocorticoid continuation contributes to the reduction in the risk for relapse. However, from the metabolic effect viewpoint, reduced glucocorticoid combined with other immunosuppressants or biological agents might be preferred.

Dialysis was an independent associated factor for the development of AS. Chronic kidney disease-mineral and bone disorder, inflammation, and hemodynamic disturbances are considered to contribute to the pathophysiology and progression of AS in dialysis patients [26]. MPO-AAV patients with AS had a significantly worse prognosis than those without AS. Therefore, strategies are needed to ameliorate the metabolic and mineral disorders in addition to preventing relapse and infection to improve MPO-AAV prognosis. Regular monitoring of echocardiography during MPO-AAV treatment is suggested.

This study had some limitations. First, the participants were MPO-AAV patients with renal comorbidities and, therefore, they are not representative of MPO-AAV patients without renal involvement. Second, this study exclusively examined a Japanese population; thus, these results may not be generalisable to other populations. Third, the cumulative dose of glucocorticoids could not be determined.

## Conclusions

The prevalence rate of AS was significantly higher in MPO-AAV patients with renal involvement at more than 1 year after the onset of vasculitis than in control CKD patients. Regular monitoring of echocardiography during MPO-AAV treatment is suggested.

## Supporting information

S1 Table Multivariable logistic regression analysis for aortic valve stenosis in 327 CKD patients in which variables are composed of MPO-AAV, dialysis dependence, age at echocardiography, sex, hypertension, and diabetes. (DOCX)

S2 Table Multivariable logistic regression analysis for aortic valve stenosis in 327 CKD patients in which variables are composed of MPO-AAV, dialysis dependence, age at echocardiography, sex, hypertension, and HDL-C. (DOCX)

S3 Table Multivariable logistic regression analysis for aortic valve stenosis in 327 CKD patients in which variables are composed of MPO-AAV, dialysis dependence, age at echocardiography, sex, hypertension, and Ca. (DOCX)

S4 Table Multivariable logistic regression analysis for aortic valve stenosis in 327 CKD patients in which variables are composed of MPO-AAV, dialysis dependence, age at echocardiography, sex, hypertension, and P. (DOCX)

S5 Table Raw data. (XLSX)
