# Supplementary material for: Development of aortic valve stenosis in myeloperoxidase antineutrophil cytoplasmic antibody-associated vasculitis with renal involvement
Source: PLoS One. 2021 Jan 22;16(1):e0245869. doi: 10.1371/journal.pone.0245869 (PMC7822555; doi:10.1371/journal.pone.0245869)
Supplement: S1 Table — (DOCX) [file pone.0245869.s001.docx]

**S1 Table.** **Multivariable logistic regression analysis for aortic valve stenosis in 327 CKD patients in which variables are composed of MPO-AAV, dialysis dependence, age at echocardiography, sex, hypertension, and diabetes.**

|  | **OR** | **95% LCI** | **95% UCI** | **p-value** |
| --- | --- | --- | --- | --- |
| MPO-AAV (yes = 1) | 2.96 | 1.55 | 5.68 | 0.001 |
| Dialysis dependence (yes = 1) | 6.61 | 3.40 | 12.85 | <0.001 |
| Age at echocardiography (per 1-year increase) | 1.00 | 0.96 | 1.05 | 0.84 |
| Sex (Male) | 1.74 | 0.91 | 3.35 | 0.096 |
| Hypertension (yes = 1) | 3.52 | 1.29 | 9.56 | 0.014 |
| Diabetes (yes = 1) | 1.96 | 0.50 | 1.82 | 0.89 |

MPO-AAV, myeloperoxidase antineutrophil cytoplasmic antibody-associated vasculitis; CKD, chronic kidney disease; OR, odds ratio; LCI, lower confidence interval; UCI, upper confidence interval.
