# Supplementary material for: Development of aortic valve stenosis in myeloperoxidase antineutrophil cytoplasmic antibody-associated vasculitis with renal involvement
Source: PLoS One. 2021 Jan 22;16(1):e0245869. doi: 10.1371/journal.pone.0245869 (PMC7822555; doi:10.1371/journal.pone.0245869)
Supplement: S2 Table — (DOCX) [file pone.0245869.s002.docx]

**S2 Table.** **Multivariable logistic regression analysis for aortic valve stenosis in 327 CKD patients in which variables are composed of MPO-AAV, dialysis dependence, age at echocardiography, sex, hypertension, and HDL-C.**

|  | **OR** | **95% LCI** | **95% UCI** | **p-value** |
| --- | --- | --- | --- | --- |
| MPO-AAV (yes = 1) | 3.18 | 1.49 | 6.78 | 0.003 |
| Dialysis dependence (yes = 1) | 6.45 | 3.27 | 12.75 | <0.001 |
| Age at echocardiography (per 1-year increase) | 1.00 | 0.96 | 1.04 | 0.87 |
| Sex (Male) | 1.71 | 0.88 | 3.32 | 0.11 |
| Hypertension (yes = 1) | 3.49 | 1.28 | 9.50 | 0.014 |
| HDL-C (yes = 1) | 0.98 | 0.98 | 1.02 | 0.75 |

MPO-AAV, myeloperoxidase antineutrophil cytoplasmic antibody-associated vasculitis; CKD, chronic kidney disease; HDL-C, high-density lipoprotein cholesterol; OR, odds ratio; LCI, lower confidence interval; UCI, upper confidence interval.
