# Supplementary material for: Development of aortic valve stenosis in myeloperoxidase antineutrophil cytoplasmic antibody-associated vasculitis with renal involvement
Source: PLoS One. 2021 Jan 22;16(1):e0245869. doi: 10.1371/journal.pone.0245869 (PMC7822555; doi:10.1371/journal.pone.0245869)
Supplement: S3 Table — (DOCX) [file pone.0245869.s003.docx]

**S3 Table. Multivariable Logistic Regression Analysis for Aortic Valve Stenosis in 327 CKD Patients in which variables are composed of MPO-AAV, dialysis dependence, age at echocardiography, sex, hypertension, and Ca**

|  | **OR** | **95% LCI** | **95% UCI** | **p-value** |
| --- | --- | --- | --- | --- |
| MPO-AAV (yes = 1) | 2.90 | 1.51 | 5.57 | 0.001 |
| Dialysis dependence (yes = 1) | 7.02 | 3.50 | 14.09 | <0.001 |
| Age at echocardiography (per 1-year increase) | 1.01 | 0.97 | 1.05 | 0.79 |
| Sex (Male) | 1.76 | 0.92 | 3.40 | 0.09 |
| Hypertension (yes = 1) | 3.50 | 1.28 | 9.54 | 0.014 |
| Ca (per 1 mg/dL increase) | 1.16 | 0.71 | 1.89 | 0.56 |

MPO-AAV, myeloperoxidase antineutrophil cytoplasmic antibody-associated vasculitis; CKD, chronic kidney disease; OR, odds ratio; LCI, lower confidence interval; UCI, upper confidence interval.
