# Supplementary material for: Development of aortic valve stenosis in myeloperoxidase antineutrophil cytoplasmic antibody-associated vasculitis with renal involvement
Source: PLoS One. 2021 Jan 22;16(1):e0245869. doi: 10.1371/journal.pone.0245869 (PMC7822555; doi:10.1371/journal.pone.0245869)
Supplement: S4 Table — (DOCX) [file pone.0245869.s004.docx]

**S4 Table. Multivariable Logistic Regression Analysis for Aortic Valve Stenosis in 327 CKD Patients**

|  | **OR** | **95% LCI** | **95% UCI** | **p-value** |
| --- | --- | --- | --- | --- |
| MPO-AAV (yes = 1) | 2.78 | 1.44 | 5.35 | 0.002 |
| Dialysis dependence (yes = 1) | 8.61 | 3.80 | 19.52 | <0.001 |
| Age at echocardiography (per 1-year increase) | 1.00 | 0.96 | 1.04 | 0.91 |
| Sex (Male) | 1.80 | 0.93 | 3.50 | 0.08 |
| Hypertension (yes = 1) | 3.64 | 1.33 | 9.97 | 0.012 |
| P (per 1 mg/dL increase) | 0.86 | 0.66 | 1.13 | 0.28 |

MPO-AAV, myeloperoxidase antineutrophil cytoplasmic antibody-associated vasculitis; CKD, chronic kidney disease; OR, odds ratio; LCI, lower confidence interval; UCI, upper confidence interval.
